# CEOs' psychological trait and firms' adoption of blockchain technology: The role of hometown identity

**DOI:** 10.3389/fpsyg.2022.1005249

**Published:** 2022-11-17

**Authors:** Liang Wang, Wenyi Xiao, Shiyu Xie, Ru Wei

**Affiliations:** ^1^College of Business, Shanghai University of Finance and Economics, Shanghai, China; ^2^School of European Languages and Cultures, Beijing Foreign Studies University, Beijing, China; ^3^International Business School, Shanghai University of International Business and Economics, Shanghai, China; ^4^College of Management Sciences, Chengdu University of Technology, Chengdu, China

**Keywords:** hometown identity, CEOs' psychological trait, psychology of identity, upper echelons theory, fintech, block-chain, China

## Abstract

The impact of CEOs' psychological traits on firms' decision-making has been explored by many psychological researchers. What's more, the CEO hometown identity, as one of the most fundamental psychological traits, has drawn increasing attention from the psychological literature. Firms' adoption of blockchain technology plays an innovative and efficient role in firms' strategic transformation. Thus, it is necessary to investigate the relationship between CEOs' psychological traits and firms' adoptions of blockchain technology from the perspective of hometown identity. To examine the impact of the CEO hometown identity on firms' adoption of blockchain technology, this paper manually collects information about the CEO hometown identity and constructs the index of firms' adoption of blockchain technology based on the textual analysis of firms' annual reports. Based on the theory about the psychology of identity, this paper constructs the theoretical hypothesis about the relationship between CEO hometown identity and firms' adoption of blockchain technology. Then, this paper uses a two-way fixed effect regression model to estimate the impact of the CEO hometown identity on firms' adoption of blockchain technology based on the panel data of Chinese A-share non-financial listed firms during 2008–2019. The research results show that: (1) the CEO hometown identity has a positive effect on firms' adoption of blockchain technology. (2) For firms with severe financing constraints and State-owned enterprises (SOEs), the positive effect of the CEO hometown identity on firms' adoption of blockchain technology is more prominent. (3) Our benchmark results still hold after a series of robustness checks, including altering the measurement of the CEO hometown identity, altering the sample, adding CEO-specific control variables, and altering the logit regression model. Based on the above-mentioned findings, this paper not only sheds new light on the power of CEOs' psychological trait but also deepens the understanding about theories of the psychology of identity.

## Introduction

Blockchain, an essential content of FinTech, has been rapidly evolving and developing (Yang and Li, 2018; Ertz and Boily, 2019). Firms' adoption of blockchain technology is an important aspect of Fin-tech development (Chen et al., 2017; Ashta and Biot-Paquerot, 2018; Kim and Sarin, 2018). Now, the bright side of firms' adoptions of blockchain technology has been explored by many scholars from multiperspectives, including the stock market performance (Chen et al., 2022), Supply chain management (Saberi et al., 2019; Deng et al., 2022), contract enforcement (Cong and He, 2019; Natanelov et al., 2022), operation efficiency (Cai and Zhu, 2016; Zheng et al., 2022), and intellectual property protection (Chen et al., 2020). Exactly, Chen et al. (2022) hold that firms' adoptions of blockchain technology improve the stock return by triggering investors' positive sentiments. Saberi et al. (2019) find that firms' adoption of blockchain technology is beneficial for sharing information and improving the stability of supply chain. Firms' adoption of blockchain technology also improves the efficiency of contract enforcement by detecting fraud in trading and smart contract (Cong and He, 2019; Natanelov et al., 2022). Therefore, firms' adoption of blockchain technology plays an innovative and efficient role in firms' strategic transformation (Rehman Khan et al., 2022). Although extant studies have revealed the positive effect of firms' adoption of blockchain technology on firm behaviors, they do not provide sufficient discussion regarding the influence factors of firms' adoption of blockchain technology.

Recently, the mainstream of literature has found that the CEO hometown identity, as one of the most fundamental psychological traits, could influence firm behaviors, such as firm innovation, investment, and tax avoidance (Shen et al., 2019; Guo et al., 2021; Ren et al., 2021a). What's more, there is no study focused on whether and how the firms' adoption of blockchain technology is affected by CEO hometown identity. This question still requires further clarification. Theories of identity psychology are extensively employed among classical studies in psychology and corporate finance, exploring the role of CEOs' psychological trait in firms' decision-making (Yonker, 2017; Kong et al., 2020; Lai et al., 2020). These theories believe that the psychology cognitive structure and inner values of CEO determine their ability to understand and interpret relevant information. Therefore, the firm's strategic decisions could be greatly affected by the psychological characteristics of CEO (Hambrick and Mason, 1984). As the makers and executors of firm strategic decisions, CEO undoubtedly would attach great significance to firms' adoptions of blockchain technology.

According to the World Intellectual Property Organization (WIPO) statistic, the number of patents related to blockchain technology increased to 5.5 ten thousand worldwide during 2012–2021. Especially, China, as the largest emerging market economy, has received and granted 3.3 ten thousand patents related to blockchain technology, which can be considered as the most competitive country in the field of blockchain technology to some extent. Many scholars hold that patents could reveal the capacity of technology innovation and adoption (Matray, 2021; Ren et al., 2021a). To some extent, the above phenomena reveal that China has a strong innovation and adoption capacity of blockchain technology.

However, Chinese listed companies not only represent the drive force in financial innovation of China (Cao et al., 2021) but also play an important role in the development of blockchain in China, including R&D (research and development) of blockchain technology and application of blockchain technology (Guo and Liang, 2016; Deng et al., 2022). For an example, Ping An Bank Co., Ltd. has been conducting cross-sectional trade credit through the blockchain technology since 2013. To illustrate the trend of blockchain technology adoption in Chinese listed companies, we calculate the frequency of occurrence of the corresponding blockchain keywords in the annual reports published by Chinese listed companies as a proxy indicator of firm adoption of blockchain technology (named *Block_chain*) and draw its distribution during 2008–2019 in Figure A1. Figure A1 shows that the adoption of blockchain technology in Chinese listed firms increases rapidly and has become an important firm in strategic decision-making.

Blockchain is an innovative technology, and the Chinese government advocates its application. Until now, China has launched a series of policies to support blockchain development. Exactly, the Chinese government explicitly advocates the development of emerging digital industries that foster blockchain and related digital industry in “The Fourteenth Five Year Plan for National Economic and Social Development of the People's Republic of China and the Outline of the Vision Goals for 2035.” Later, “Guiding Opinions on Accelerating the Application of blockchain Technology and Industrial Development” was jointly launched by the Chinese Ministry of Industry and Information Technology and CPC Central Cyber Information Office in 2021. In addition, the Chinese government also establishes a blockchain intellectual property protection workstation in Shenzhen city, which aims to foster blockchain development. Therefore, the Chinese institutional context provides an ideal environment for our research.

The CEO hometown identity, as a vital psychological feature, its effects and implications have been understudied. In this study, we pay particular attention to the effect of CEO hometown identity on firms' adoption of blockchain technology. The CEO hometown identity is one of the hidden characteristics, which subtly influence the CEO's thoughts and behaviors. Additionally, this feature reflects the emotional link between individuals and their hometowns. Previous studies have found that the CEO hometown identity has a positive effect on firm behaviors, especially in the fields of corporate innovation (Lai et al., 2020; Ren et al., 2021b), corporate investment opportunities (Pool et al., 2012; Kong et al., 2020; Guo et al., 2021), and employee benefits (Yonker, 2017). Extant studies do not provide sufficient discussions regarding the influence factors of firms' adoption of blockchain technology, and papers extending home identity theory to explain the effect of in-group favoritism on firms' adoptions of blockchain technology are still scarce. Will the local CEO be affected by the hometown identity and engage in more adoptions of blockchain technology? This paper aims to fill this research void.

Based on the psychology of identity's theories (Glover, 1988; Hammack, 2008), we propose that the CEO hometown identity is positively related to firms' adoptions of blockchain technology for the following reasons: First, local CEOs have a natural identity and attachment to their hometown, then they can push the firms' adoptions of blockchain technology which contribute to the development of local fintech. What's more, Hometown identity will let CEOs be more concerned about the corporate and individual reputation in their hometown, and then focus on the fulfillment of firms' adoptions of blockchain technology and contribute to the development of fintech in hometown. Additionally, as part of group members, the local CEOs will take more care about the evaluation from hometown, especially when the investment object of blockchain technology is related to the place of registration. Therefore, local CEOs will reduce their opportunistic behaviors for personal gains and improve the performance of the blockchain technology adoption based on hometown identity psychology.

To investigate the relationship between CEO hometown identity and firms' adoption of blockchain technology, we hand-collect information about the CEO hometown identity among Chinese listed firms during 2008–2019 and form a unique dataset about CEOs' hometown identity, which provide evidence to define whether CEOs' hometown is consistent with the location of his (her) affiliated firm. Then, we follow related literature (Tian et al., 2022; Wu et al., 2022) and construct a measurement of firms' adoption of blockchain technology-based textual analysis of Chinese listed firms' annual report during 2008–2019. Based on the above works, we employ a two-way fixed effect regression model to estimate the effect of the CEO hometown identity on firms' adoption of blockchain technology based on the panel data of Chinese A-share non-financial listed firms during 2008–2019. According to our empirical results, we find that: (1) the CEO hometown identity has a positive effect on firms' adoption of blockchain technology. (2) For firms with severe financing constraints and SOEs, the positive effect of the CEO hometown identity on firms' adoption of blockchain technology is more prominent. (3) Our benchmark results still hold after a series of robustness checks, including altering the measurement of the CEO hometown identity, altering the sample, adding CEO-specific control variables, and altering the logit regression model.

This study contributes to the CEO hometown identity and firms' adoptions of blockchain technology literature in three ways. First, this study enriches the effect of CEO's psychological features on corporate strategic decision-making and defines hometown identity as “the CEO's birthplace is consistent with the firm's registered place.” It can help us to have a deeper understanding on how the psychological bias of CEO can influence the corporate strategic selection and economic outcomes (Shen et al., 2019; Kong et al., 2020; Lai et al., 2020; Ren et al., 2021a). Second, this study advances the investigation on the impact of CEO's psychological feature about hometown identity on firms' adoptions of blockchain technology from the perspective of the firms' adoptions of blockchain technology. This study expands the understanding of the factors affecting the firms' adoptions of blockchain technology and breaks through the previous studies based on the single perspective of the corporate characteristics (Tian et al., 2022; Wu et al., 2022).

The remainder of this paper is structured as follows: Section Literature review and hypothesis development presents the literature and hypothesis development. Section Research design and methodology describes the empirical strategy that includes empirical models, sample selection, and variables. Section Benchmark empirical results provides benchmark results and discussion. Section Robust check conducts a series of robustness checks. Section Heterogeneous analyses focuses on heterogeneous analyses. Section Conclusion concludes conclusions and insights.

## Literature review and hypothesis development

### CEO hometown identity

In our study, the hometown identity refers to psychological preferences that individuals are likely to form a favoritism when making decisions since emotional attachment, which might be affected by traditional Chinese concepts, such as “returning to the hometown” and “difficult to leave the homeland.” Blake (1974) first came up with the definition of topophilia as a person's emotional attachment to the environment, that is, the spiritual, emotional, and cognitive bond with a certain place. Proshansky (1978) put forward the concept of place identity based on the place attachment, which is the interaction that exists in the personal consciousness, including thoughts, beliefs, preferences, and values.

Prior research suggests that individual decisions may be affected by the hometown identity mainly due to various reasons, such as favoritism, associated advantages, etc. For example, Fund managers with more home bias would acquire premium through social networks and information advantages (Pool et al., 2012), and local CEOs can significantly increase the opportunity to obtain trade credits based on the channel of information change and social trust (Kong et al., 2020). The CEO hometown identity has a positive and significant influence on firm innovation. Financial officials prefer their hometown when allocating financial resources (Yin and Chen, 2021). After getting support from the boards, the local CEOs would like to take on more risks and have the longing plans to develop R and D activities (Ren et al., 2021b). However, existing research remains silent on the effect of hometown identity on firms' adoptions of blockchain technology. Therefore, we try to fill this research gap.

### CEO hometown identity and firms' adoptions of blockchain technology

We defined the local CEO, as the birthplace of the CEO is the same as the registered place of the firm for which they work. From the psychological perspective of identity, local CEOs at least have the dual identity of a position and a fellow. CEOs influenced by the “fellow identity” would be constrained by the social psychological moral opinion, which can encourage local CEOs to do some action to help in the development of their hometown. Blockchain technology is an innovative technology that helps in the development of fintech. Therefore, local CEOs will accelerate the firms' adoption of blockchain technology, which aims to boost local fintech. What's more, Hometown identity will let CEOs be more concerned about the corporate and individual reputation in their hometown and then focus on fulfilling firms' adoptions of blockchain technology and contributing to the development of fintech in their hometown.

Additionally, as part of group members, local CEOs will care more about the evaluation from their hometown, especially when the investment object of blockchain technology is related to the firms' location. Therefore, local CEOs will reduce their opportunistic behaviors for personal gains and improve the performance of the blockchain technology adoption based on hometown identity psychology. Thus, we have our main hypothesis:

Hypothesis: CEO hometown identity has a positive effect on firms' adoptions of blockchain technology.

## Research design and methodology

### Data source

We select all Chinese non-financial public companies listed on the Shanghai and Shenzhen exchanges from 2008 to 2019. The data of listed companies were obtained from China Stock Market and Accounting Research (CSMAR) database. The hometown identity of CEO was hand-collected from Sina Finance figures, Google, Baidu, Bing, and other online searches. Then, we obtain raw sample that includes 6803 firm-year observations. Following previously related research (Kong et al., 2020; Wu et al., 2022), we impose the following restrictions: (1) we delete firms from the financial and real estate industries; (2) we remove firms with missing tandem mass tag (TMT) information; (3) we exclude observations with insufficient data for calculating financial and corporate governance variables; and (4) we delete firms that were specially treated. Our final sample includes 5,534 firm-years. This study winsorizes the continuous variables at the 1 and 99% levels to minimize outlier effects.

### Variable description

#### The CEO hometown identity (local CEO)

Our study chose the sample from listed Chinese firms from 2008 to 2019. First, the CEO's hometown data were based on the executive resumes and hometown information disclosed in the China Stock Market and Accounting Research (CSMAR) database. We combined Sina Finance figures, Google, Baidu, Bing, and other online searches and manually collected and sorted them. We define the CEO as a local CEO when the CEO's hometown is located in the same province as the firm's province, and the variable *Local*_*CEO* takes the value of 1, and *Local*_*CEO* takes the value of 0 otherwise.

#### Firms' adoptions of blockchain technology measures

The quantitative measurement of firms' adoptions of blockchain technology is a frontier question in academic. At present, mainstream literature on firms' adoptions of blockchain technology is based on theoretical qualitative analysis (Cong and He, 2019; Feng et al., 2020; Deng et al., 2022). In contrast, quantitative research based on firms' adoptions of blockchain technology is rare. To empirically test the influence factors of firms' adoptions of blockchain technology, we need to carefully capture the firms' behavioral variation of “adoptions of blockchain technology.” Some scholars have made beneficial attempts in quantitative analysis, such as Tian et al. (2022) and Wu et al. (2022), who calculate the frequency of keywords related to digital technology which occurs in the annual report of listed firms as the measurement of digital transformation. What's more, the number of keywords related to blockchain technology occurs in the annual report of listed firms that contain the firms' preferences, attitudes, and action statements for blockchain technology adoption. Therefore, it is feasible and scientific to construct the measurement of firms' adoption of blockchain technology based on the frequency of keywords related to blockchain in the annual reports of the listed firm.

The measurement of firms' adoptions of blockchain technology is based on the textual analysis method. Now, extant literature a referring to the existing literature (Tian et al., 2022; Wu et al., 2022), we calculate the frequency of occurrence of the corresponding blockchain keywords in the annual reports published by listed companies as a proxy indicator (named *Block_chain*) for firms' adoption of blockchain technology.

#### Control variables

To control lots of factors that might potentially affect firms' adoption of blockchain technology, we included some related controls in our study. Details can be described as follows.

Firm size (*Size*), the natural logarithm of total assets at the end of the period, was used for this measurement. Firm leverage (*Lev*), we use current year's asset–liability ratio to measure the firm leverage. Book-to-market value (*BM*), book-to-market value was used for this measurement. Return on Asset (*ROA*), return on asset was used for this measurement. Number of board members (*Board*), number of board members was used for this measurement. The ratio of shares held by the largest shareholder (%) and named *Top1*, the ratio of shares held by the largest shareholder was used for this measurement. The number of independent board director members divided by the number of board director members (%) and named *Indep*, the number of independent board director members divided by the number of board director members (%) were used for this measurement. State-owned enterprise (*SOE*), State-owned enterprise is a dummy variable that is equal to 1, if the firm is a State-own enterprise, and equal to 0 otherwise.

### Model methodology

To test our hypothesis, we establish the following benchmark regression model:


(1) $$\begin{array}{l} Block\text{\_}chain_{i,t}=F_{0}+F_{1}Local\text{\_}CEO_{i,t}+F_{2}CV_{i,t}+Year\\ +Ind+c_{i,t} \end{array}$$

Where i denotes firms, t denotes time, and *Block_chain*_*i,t*_ denotes firms' adoption of blockchain technology. *Local_CEO*_*i,t*_ denotes the CEO hometown identity. *CV*_*i,t*_ is the set of control variables for firm characteristics. *c*_*i,t*_ is the error term. As we stated in the main hypothesis, a significant positive estimate of *F*_1_ will support our prediction. *Year* denotes year fixed effect and *Ind* denotes industry fixed effect.

## Benchmark empirical results

### Summary statistic

Table 1 presents the results of descriptive statistics for the main variables. According to the results of Table 2, the maximum value of *Block_chain* is 23 and the minimum value is 0, indicating that there are large variations in the level of adoption of blockchain technology among different sample firms. Therefore, the relationship between *Block_chain* and *Local_CEO* needs to be further investigated. In addition, the proportion of firms without adoption of the blockchain technology was 54.63% during 2008–2019. The 25% Percentile of *Block_chain* is 0. The 75% Percentile of *Block_chain* is 1.

**Table 1:** Descriptive statistics of the main variables.

**Variables**	***N***	**Mean**	**SD**	**Min**	**Median**	**Max**
*Block_chain*	5,534	0.518	1.015	0.000	0.000	23.000
*Local_CEO*	5,534	0.597	0.491	0.000	1.000	1.000
*MB*	5,534	0.567	0.265	0.032	0.540	1.407
*ROA*	5,534	0.045	0.067	−1.130	0.040	0.477
*Lev*	5,534	0.450	0.208	0.008	0.451	1.173
*Size*	5,534	23.245	1.438	19.827	23.042	29.509
*Board*	5,534	8.858	1.902	4.000	9.000	18.000
*Top1*	5,534	36.380	15.324	2.197	34.410	86.350
*Indep*	5,534	37.550	5.905	14.290	35.500	80.000
*SOE*	5,534	0.451	0.498	0.000	0.000	1.000

**Table 2:** Correlation coefficient matrix.

**Variables**	***Block_chain***	***Local_CEO***	***MB***	***ROA***	***Lev***	***Size***	***Board***	***Top1***	***Indep***	***SOE***
*Block_chain*	1	−0.051^***^	0.014	−0.002	−0.024*	0.049^***^	−0.048^***^	−0.060^***^	0.052^***^	−0.066^***^
*Local_CEO*	0.004	1	0.018	0.044^***^	−0.051^***^	−0.072^***^	0.004	−0.040^***^	−0.049^***^	−0.020
*MB*	0.006	0.013	1	−0.376^***^	0.496^***^	0.591^***^	0.154^***^	0.096^***^	0.001	0.328^***^
*ROA*	−0.002	0.040^***^	−0.275^***^	1	−0.395^***^	−0.053^***^	0.008	0.080^***^	−0.014	−0.167^***^
*Lev*	−0.007	−0.044^***^	0.478^***^	−0.337^***^	1	0.520^***^	0.135^***^	0.053^***^	0.009	0.302^***^
*Size*	0.038^***^	−0.112^***^	0.603^***^	−0.015	0.505^***^	1	0.204^***^	0.188^***^	0.057^***^	0.349^***^
*Board*	−0.009	0.006	0.173^***^	0.024*	0.138^***^	0.252^***^	1	0.017	−0.465^***^	0.306^***^
*Top1*	−0.046^***^	−0.046^***^	0.102^***^	0.098^***^	0.057^***^	0.231^***^	0.036^***^	1	0.054^***^	0.137^***^
*Indep*	0.013	−0.066^***^	0.014	0.004	0.009	0.089^***^	−0.396^***^	0.058^***^	1	−0.077^***^
*SOE*	−0.061^***^	−0.020	0.333^***^	−0.084^***^	0.299^***^	0.356^***^	0.312^***^	0.137^***^	−0.068^***^	1

This table reports the correlation coefficients between the variables, the lower triangular matrix reports the Pearson correlation coefficient and the upper triangular matrix reports the Spearman correlation coefficient. The variable definitions are shown in this table.

*** ,

** , and

* represent the 1, 5, and 10% significance levels, respectively.

### Correlation analysis

Table 2 shows the correlation coefficients of the main variables. According to Table 2, it can be found that the correlation coefficients between most variables are <0.5, indicating that the problem of multicollinearity between explanatory variables is not serious.

### Benchmark regression: CEO hometown identity and firms' adoption of blockchain technology

Table 3 provides the regression estimation results of the hypothesis. Column (1) in Table 3 shows the estimation results controlling for industry- and year fixed effects, while column (2) shows the estimation results controlling for both industry-, year fixed effects, and control variables.

**Table 3:** Benchmark regression results.

	**(1)**	**(2)**
**Variables**	***Block_chain***	***Block_chain***
*Local_CEO*	0.054^**^	0.067^**^
	(0.026)	(0.027)
*MB*		−0.103
		(0.078)
*ROA*		−0.046
		(0.217)
*Lev*		−0.006
		(0.083)
*Size*		0.058^***^
		(0.015)
*Board*		0.013
		(0.008)
*Top1*		−0.002^**^
		(0.001)
*Indep*		0.001
		(0.002)
*SOE*		−0.102^***^
		(0.031)
Constant	0.057^***^	−1.263^***^
	(0.020)	(0.302)
Industry fix effect	Yes	Yes
Year fix effect	Yes	Yes
N	5,534	5,534
R^2^	0.067	0.072

Standard errors are in parentheses,

*** ,

** , and

* represent the 1, 5, and 10% significance levels, respectively.

From the estimation results in column (1) of Table 3, the estimated coefficient of *Local_CEO* is significantly positive at 5% level without control variables. The results in column (2) indicate that the estimated coefficient of *Local_CEO* remains significantly positive (0.067) at 5% level with control variables. All the above results support the hypothesis that the CEO hometown identity has a positive effect on firms' adoption of blockchain technology.

Based on the estimation result in Column 2 of Table 3, firms with hometown CEOs, on average, increase the frequency of occurrence of block-chain keywords in annual reports by 0.067 times more than similar firms with non-hometown CEOs. Considering that the average frequency of occurrence of block-chain keywords in annual reports is 0.518 times, this effect is also economically significant. On the one hand, the distribution of the frequency of occurrence of block-chain keywords in annual reports is largely skewed to the right. On the other hand, the frequency of occurrence of block-chain keywords in annual reports is a count variable. Therefore, we also employ the logit model to estimate the coefficient of *Local_CEO* in robustness checks and find that the coefficient is 0.664 (significant in 1%), which also verifies that the effect of *Local_CEO* is economically significant. What's more, Ren et al. (2021a) hold that firm with a hometown CEO, the R and D intensity of a firm is, on average, 0.086% higher than a firm with a non-hometown CEO. Considering that the average R&D intensity is 1.12%, this effect is also economically significant.

## Robust check

### Lag of local CEO

First, the *Local_CEO* variable is lagged by one period and the estimation results are shown in column (1) and column (2) of Table 4. The lagged one-period *Local_CEO* variable (*Local_CEO_Lag*) is significantly positive at the 5% level.

**Table 4:** Robustness check: Lag of local CEO.

	**(1)**	**(2)**
	***Block_chain***	***Block_chain***
*Local_CEO_Lag*	0.077^**^	0.095^***^
	(0.032)	(0.032)
*MB*		−0.138
		(0.096)
*ROA*		0.072
		(0.272)
*Lev*		0.106
		(0.104)
*Size*		0.068^***^
		(0.018)
*Board*		0.019^**^
		(0.010)
*Top1*		−0.002
		(0.001)
*Indep*		0.001
		(0.003)
*SOE*		−0.136^***^
		(0.037)
*Constant*	0.046^*^	−1.607^***^
	(0.024)	(0.369)
Industry fix effect	Yes	Yes
Year fix effect	Yes	Yes
N	4,173	4,173
R^2^	0.072	0.081

Standard errors are in parentheses,

*** ,

** , and

* represent the 1, 5, and 10% significance levels, respectively.

Based on the above empirical results, it can be found that CEO hometown identity has a positive effect on firms' adoption of blockchain technology after lagging one-period *Local_CEO* variable (*Local_CEO_Lag*) for regression. Therefore, our benchmark results are robust.

### Alternative measurement of local CEO

We redefine the CEO as a local CEO when a CEO's birthplace is at the same city as the firm's registered city, and the variable *Local*_*CEO_City* takes the value of 1, and *Local*_*CEO_City* takes the value of 0 otherwise. The sample size of local CEO at city level is consistent with the sample size of local CEO at province level, because we hand-collect CEOs' hometown information containing province and city simultaneously. The estimation results are shown in column (1) and column (2) of Table 5.

**Table 5:** Robustness check: Altering the measurement of local CEO.

	**(1)**	**(2)**
**Variables**	***Block_chain***	***Block_chain***
*Local_CEO_City*	0.052^**^	0.064^**^
	(0.026)	(0.027)
*MB*		−0.101
		(0.078)
*ROA*		−0.043
		(0.217)
*Lev*		−0.006
		(0.083)
*Size*		0.057^***^
		(0.015)
*Board*		0.013
		(0.008)
*Top1*		−0.002^**^
		(0.001)
*Indep*		0.001
		(0.002)
*SOE*		−0.101^***^
		(0.031)
*Constant*	0.058^***^	−1.252^***^
	(0.020)	(0.302)
Industry fix effect	Yes	Yes
Year fix effect	Yes	Yes
N	5,534	5,534
R^2^	0.067	0.072

Standard errors are in parentheses,

*** ,

** , and

* represent the 1, 5, and 10% significance levels, respectively.

Based on the above empirical results, it can be found that the CEO hometown identity has a positive effect on firms' adoption of blockchain technology after altering the measurement of *Local_CEO*. Therefore, our benchmark results are robust.

### Other robustness checks

First, considering the fact that the market penetration rate of block-chain technology increased from 0.27 to 0.64% from 2017 to 2019, we conduct robust check by estimating the benchmark regression model based on the sample in the period 2017–2019 to exclude the years when neither local nor non-local CEOs embraced blockchain technology. We reported related results in Table A1. Then, we find our benchmark findings still hold.

Second, considering that the frequency of Blockchain is associated with the probability of firm adoption of technology, we employ a logit regression model to test our benchmark regression model. Then, related results are reported in Table A2 and are consistent with our benchmark finding.

Third, due to CEO-specific variables correlated with CEO behaviors, we add some important CEO-specific control variables, including CEO age (*CEO_age*), CEO gender (*CEO_gender*), CEOs' duality (equal to 1 if CEO is chairman, and equal to 0 otherwise; the related variable name is *CEO_dualit*y), and CEO shareholding (*CEO_share*) into our benchmark regression model. Then, we report related results in Table A3 and find our benchmark results still robust.

In addition, the variable of firms' adoption of block-chain is largely skewed to the right. We also calculated the Natural logarithm of (*Block_chain*+1) and employ it in our benchmark regression as a robustness check of alternative measurements. We reported related results in Table A4. After conducting a robustness check of alternative measurements, we find that our benchmark results still hold.

## Heterogeneous analyses

The above empirical results have proved the relationship between the CEO hometown identity and firms' adoption of blockchain technology. This section further examines the moderating effect of State-owned enterprises (SOEs) and financing constraints on the relationship between the CEO hometown identity and firms' adoption of blockchain technology.

### State-owned enterprises

According to our calculation, the proportion of local CEOs in SOE is 58.61%. The proportion of local CEOs in non-SOE is 60.63%. The proportion of adoptions of block-chain technology in SOE is 44.15%. The proportion of adoptions of block-chain technology in non-SOE is 46.38%. Therefore, SOEs play a potential heterogeneous role in the relationship between CEO hometown identity and firms' adoptions of block-chain technology. It is necessary to conduct subsample regression based on the SOEs. Related results are shown in Table 6.

**Table 6:** Test the effect of State-owned enterprises (SOEs).

	**(1)**	**(2)**
**Variables**	**SOEs** ***Block_chain***	**Non-SOEs** ***Block_chain***
*Local_CEO*	0.085^***^	0.015
	(0.031)	(0.052)
*MB*	−0.095	−0.139
	(0.091)	(0.151)
*ROA*	0.257	−1.182^***^
	(0.255)	(0.408)
*Lev*	0.042	−0.218
	(0.100)	(0.152)
*Size*	0.054^***^	0.102^***^
	(0.017)	(0.033)
*Board*	0.018^*^	−0.006
	(0.010)	(0.014)
*Top1*	−0.002^*^	−0.003^*^
	(0.001)	(0.002)
*Indep*	0.002	−0.005
	(0.003)	(0.004)
*SOE*	−0.121^***^	−0.132^**^
	(0.037)	(0.066)
*Constant*	−1.299^***^	−1.638^**^
	(0.352)	(0.676)
Industry fix effect	Yes	Yes
Year fix effect	Yes	Yes
N	4,465	1,069
R^2^	0.068	0.138

Standard errors are in parentheses,

*** ,

** , and

* represent the 1, 5, and 10% significance levels, respectively.

According to Table 6, we find that the positive effect of CEO hometown identity on firms' adoption of blockchain technology is significant when the firms belong to the subgroup of SOEs.

### Financing constraint

Based on the approach of Kaplan and Zingales (1997), the measurement method is quantified by using the absolute value to indicate the degree of financing constraint of KZ index. The greater the value is, the greater the corresponding financing constraint is. In this paper, samples were ranked by using the median log value of the absolute value of the KZ index and generating a dummy named *FC* equal to 1 in case of sample data with higher financing constraints and otherwise. Then, we divide the samples into two groups of sample data with higher financing constraints (FC_High) and lower financing constraint (FC_Low).

According to Table 7, we find that the positive effect of the CEO hometown identity on firms' adoption of blockchain technology is more significant and prominent when there are firms with higher financing constraints. Then, to verify the robustness of above finding, we add the interaction term “*Local_CEO*×*FC*” into our benchmark model and report the estimation results in Table A5. According to Table A5, we find that the coefficient of interaction term “*Local_CEO*×*FC*” is significantly positive in 5%, which also indicates that the effect of the CEO hometown identity on firms' adoption of blockchain technology is more significant and prominent when there are firms with higher financing constraints.

**Table 7:** Test the effect of financing constraint.

	**(1)**	**(2)**
	**FC_High** ***Block_chain***	**FC_Low** ***Block_chain***
*Local_CEO*	0.071^**^	0.069^*^
	(0.036)	(0.041)
*MB*	−0.227^**^	−0.003
	(0.107)	(0.126)
*ROA*	0.088	−0.018
	(0.283)	(0.403)
*Lev*	0.058	−0.103
	(0.125)	(0.146)
*Size*	0.074^***^	0.045^*^
	(0.020)	(0.023)
*Board*	0.017^*^	0.010
	(0.010)	(0.013)
*Top1*	−0.001	−0.003^**^
	(0.001)	(0.001)
*Indep*	0.002	−0.000
	(0.003)	(0.004)
*SOE*	−0.095^**^	−0.107^**^
	(0.040)	(0.051)
*Constant*	−1.698^***^	−0.871^*^
	(0.401)	(0.487)
Industry fix effect	Yes	Yes
Year fix effect	Yes	Yes
N	2,810	2,724
R^2^	0.093	0.074

Standard errors are in parentheses,

*** ,

** , and

* represent the 1, 5, and 10% significance levels, respectively.

## Conclusions

The hometown identity is a vital common sense of humanity. Nowadays, firms' adoption of blockchain technology in Chinese becomes the important driven force of financial innovation and economic development. The impact of CEOs' psychological traits on firms' decision-making is a hot topic in academia. What's more, Firms' adoption of blockchain technology plays an innovative and efficient role in firms' strategic transformation. Therefore, it is vital to explore the relationship between CEOs' psychological trait and firms' adoptions of blockchain from the perspective of hometown identity. To investigate the effect of CEO hometown identity on firms' adoption of blockchain technology, this paper manually collects information about CEO hometown identity and constructs the index of firms' adoption of blockchain technology based on the textual analysis of firms' annual report. Based on the theory about the psychology of identity, this paper constructs the theoretical hypothesis about the relationship between CEO hometown identity and firms' adoption of blockchain technology. As for empirical work, this paper uses a two-way fixed effect regression model to estimate the impact of the CEO hometown identity on firms' adoption of blockchain technology based on the panel data of Chinese A-share nonfinancial listed firms during 2008–2019. Based on our empirical results, we find that: (1) the CEO hometown identity has a positive effect on firms' adoption of blockchain technology. (2) For firms with severe financing constraints and SOEs, the positive effect of the CEO hometown identity on firms' adoption of blockchain technology is more prominent. (3) Our benchmark results still hold after a series of robustness checks, including altering the measurement of the CEO hometown identity, altering the sample, adding CEO-specific control variables, and altering the logit regression model.

Based on the above research work and findings, this paper not only sheds new light on the power of CEOs' psychological traits but also deepens the understanding of theories about the psychology of identity. In the future, the CEO hometown identity, as one of the most fundamental psychological traits, will draw increasing attention from psychological literature and corporate finance literature. The consequence of CEO hometown identity, especially on fintech transformation related to firms' strategic decision-making, needs to be taken more into consideration. With the background that blockchain technology is still rising and boosting worldwide, firms play an important role in the invention, adoption, and application of blockchain technology. So, what firm-specific factors can drive the development of blockchain technology? This question still needs further exploration from the firm's financial performance, corporate governance, and so on, which help policymakers supervise and guide the development of blockchain technology.

## Data availability statement

The original contributions presented in the study are included in the article/Supplementary material, further inquiries can be directed to the corresponding author/s.

## Author contributions

All authors listed have made a substantial, direct, and intellectual contribution to the work and approved it for publication.

## Funding

This work was supported by the 2021 Postgraduate Innovation Funds for the Shanghai University of Finance and Economics (grant numbers CXJJ-202-404).

## Conflict of interest

The authors declare that the research was conducted in the absence of any commercial or financial relationships that could be construed as a potential conflict of interest.

## Publisher's note

All claims expressed in this article are solely those of the authors and do not necessarily represent those of their affiliated organizations, or those of the publisher, the editors and the reviewers. Any product that may be evaluated in this article, or claim that may be made by its manufacturer, is not guaranteed or endorsed by the publisher.
