# Supplementary material for: CEOs' psychological trait and firms' adoption of blockchain technology: The role of hometown identity
Source: Front Psychol. 2022 Nov 17;13:1005249. doi: 10.3389/fpsyg.2022.1005249 (PMC9713813; doi:10.3389/fpsyg.2022.1005249)
Supplement: Supplementary file 1 [file Data_Sheet_1.docx]

**Appendix A:**

Figure A1. *Block_chain* distribution during 2008-2019

Table A1: Robustness check based on the sample in period 2017-2019

|  | (1) | (2) |
| --- | --- | --- |
| Variables | *Block_chain* | *Block_chain* |
| *Local_CEO* | 0.285^***^ | 0.290^***^ |
|  | (0.098) | (0.098) |
| Constant | 0.148^**^ | -2.642^**^ |
|  | (0.071) | (1.091) |
| Controls | Yes | Yes |
| Industry Fix effect | Yes | Yes |
| Year Fix effect | Yes | Yes |
| N | 1457 | 1457 |
| R^2^ | 0.133 | 0.141 |
| Notes: Standard errors are in parenthesis, *^***^ p<0.01, ^**^ p<0.05, ^*^ p<0.1* | | |

Table A2: Robustness check based on logit model

|  | (1) | (2) |
| --- | --- | --- |
| Variables | *Block_chain* | *Block_chain* |
| *Local_CEO* | 0.664^***^ | 0.576^***^ |
|  | (0.176) | (0.183) |
| Constant | -3.344^***^ | -14.573^***^ |
|  | (0.117) | (1.816) |
| Controls | Yes | Yes |
| Industry Fix effect | Yes | Yes |
| Year Fix effect | Yes | Yes |
| N | 5534 | 5534 |
| Pseudo R^2^ | 0.332 | 0.365 |
| Notes: Standard errors are in parenthesis, *^***^ p<0.01, ^**^ p<0.05, ^*^ p<0.1* | | |

Table A3: Robustness check based on adding CEO-specific control variables

|  | (1) | (2) |
| --- | --- | --- |
| Variables | *Block_chain* | *Block_chain* |
| *Local_CEO* | 0.064^**^ | 0.077^***^ |
|  | (0.028) | (0.029) |
| *CEO_age* | 0.004^**^ | 0.004^*^ |
|  | (0.002) | (0.002) |
| *CEO_dualit*y | -0.047 | -0.071^**^ |
|  | (0.030) | (0.032) |
| *CEO_share* | 0.000 | -0.000^*^ |
|  | (0.000) | (0.000) |
| *MB* |  | -0.123 |
|  |  | (0.085) |
| *ROA* |  | -0.050 |
|  |  | (0.232) |
| *Lev* |  | -0.022 |
|  |  | (0.090) |
| *Size* |  | 0.065^***^ |
|  |  | (0.017) |
| *Board* |  | 0.011 |
|  |  | (0.009) |
| *Top1* |  | -0.002^**^ |
|  |  | (0.001) |
| *Indep* |  | 0.002 |
|  |  | (0.003) |
| *SOE* |  | -0.149^***^ |
|  |  | (0.036) |
| Constant | -0.144 | -1.587^***^ |
|  | (0.110) | (0.344) |
| Industry Fix effect | Yes | Yes |
| Year Fix effect | Yes | Yes |
| N | 5084 | 5084 |
| R^2^ | 0.065 | 0.072 |
| Notes: Standard errors are in parenthesis, *^***^ p<0.01, ^**^ p<0.05, ^*^ p<0.1* | | |

Table A4: Robustness check based on the log of (*Block_chain*+1*)*

|  | (1) | (2) |
| --- | --- | --- |
| Variables | *ln (Block_chain+1)* | *ln(Block_chain+1)* |
| *Local_CEO* | 0.013^***^ | 0.009^***^ |
|  | (0.003) | (0.002) |
| Constant | 0.028^***^ | -0.359^***^ |
|  | (0.005) | (0.069) |
| Controls | Yes | Yes |
| Industry Fix effect | Yes | Yes |
| Year Fix effect | Yes | Yes |
| N | 5534 | 5534 |
| R^2^ | 0.113 | 0.121 |
| Notes: Standard errors are in parenthesis, *^***^ p<0.01, ^**^ p<0.05, ^*^ p<0.1* | | |

Table A5: Test the moderate effect of financing constraints by estimating interaction term

|  | (1) | (2) |
| --- | --- | --- |
| Variables | *Block_chain* | *Block_chain* |
| *Local_CEO* | 0.061^**^ | 0.068^**^ |
|  | (0.031) | (0.032) |
| *Local_CEO×FC* | 0.009^**^ | -0.011^**^ |
|  | (0.004) | (0.005) |
| Constant | 0.057^***^ | -1.262^***^ |
|  | (0.020) | (0.303) |
| Controls | Yes | Yes |
| Industry Fix effect | Yes | Yes |
| Year Fix effect | Yes | Yes |
| N | 5534 | 5534 |
| R^2^ | 0.067 | 0.072 |
| Notes: Standard errors are in parenthesis, *^***^ p<0.01, ^**^ p<0.05, ^*^ p<0.1* | | |
